# Supplementary material for: Phytochemical, Morphological, and Physiological Variation in Different Ajowan (Trachyspermum ammi L.) Populations as Affected by Salt Stress, Genotype × Year Interaction and Pollination System
Source: Int J Mol Sci. 2023 Jun 21;24(13):10438. doi: 10.3390/ijms241310438 (PMC10341850; doi:10.3390/ijms241310438)
Supplement: Supplementary file 1 [file ijms-24-10438-s001.zip › ijms-2421156-SI.pdf]

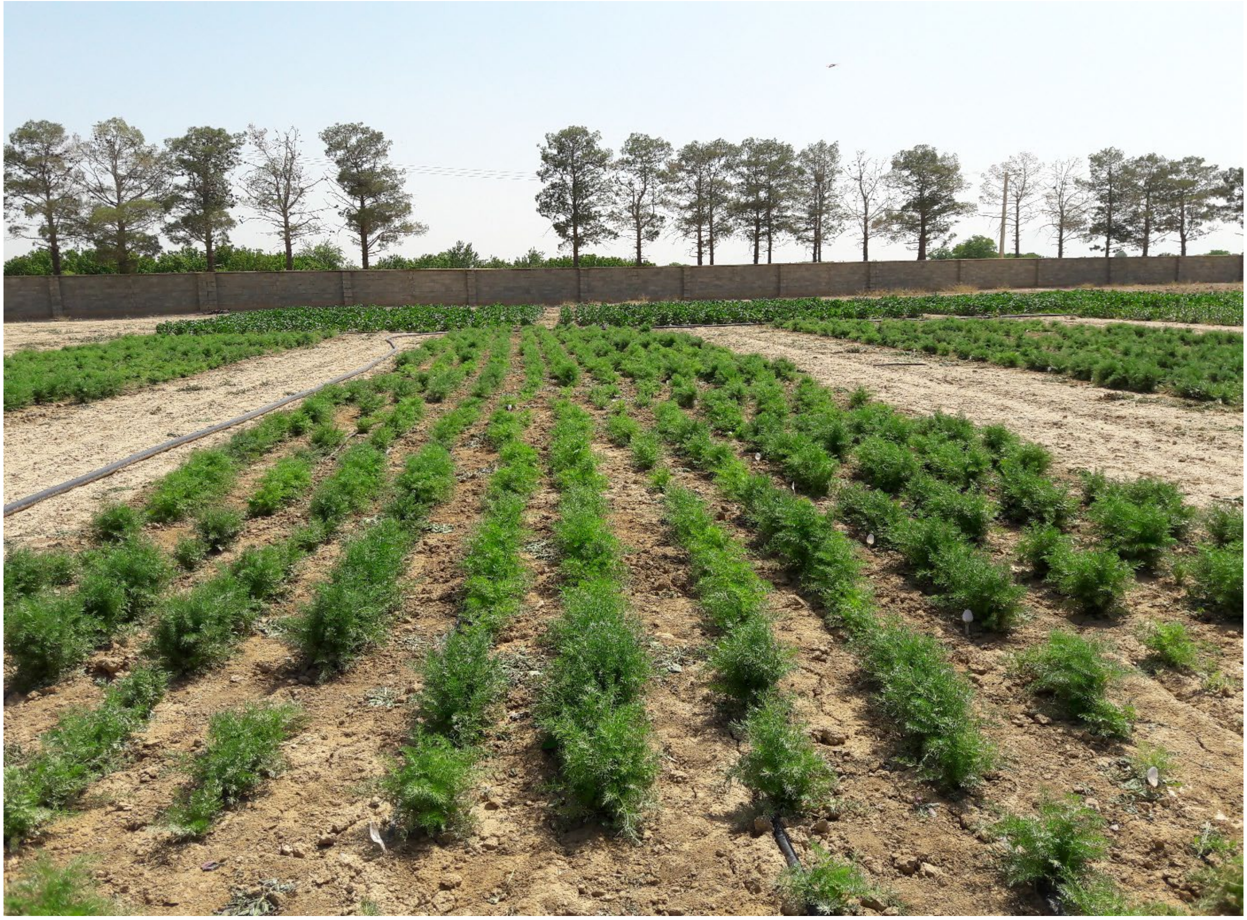

(**a**)

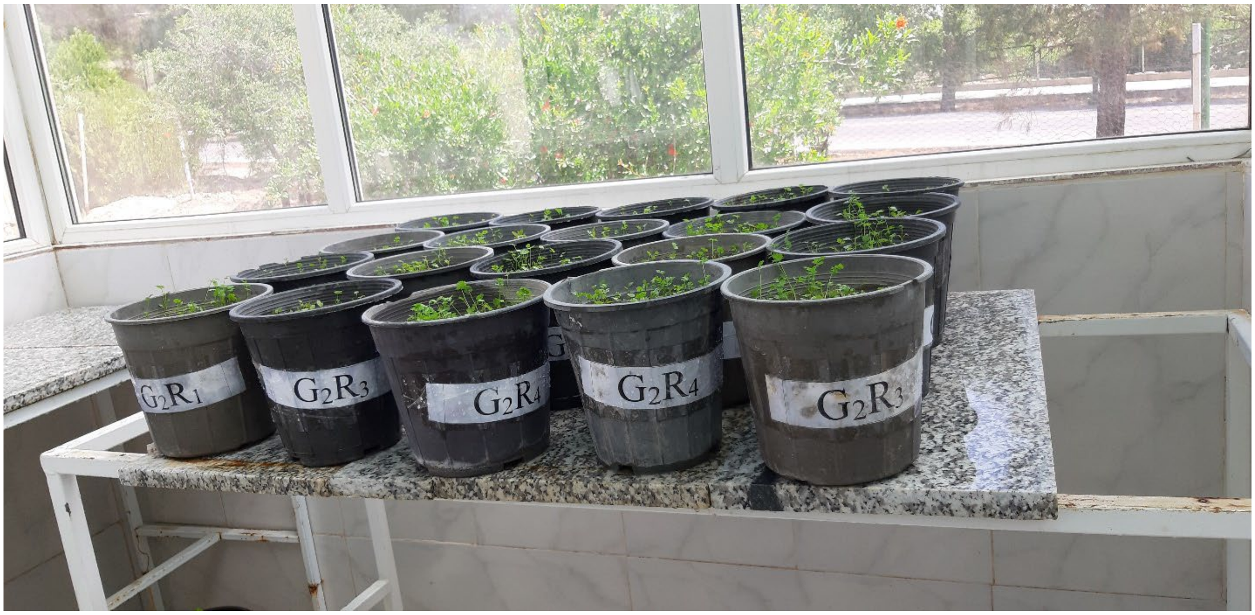

(**b**)

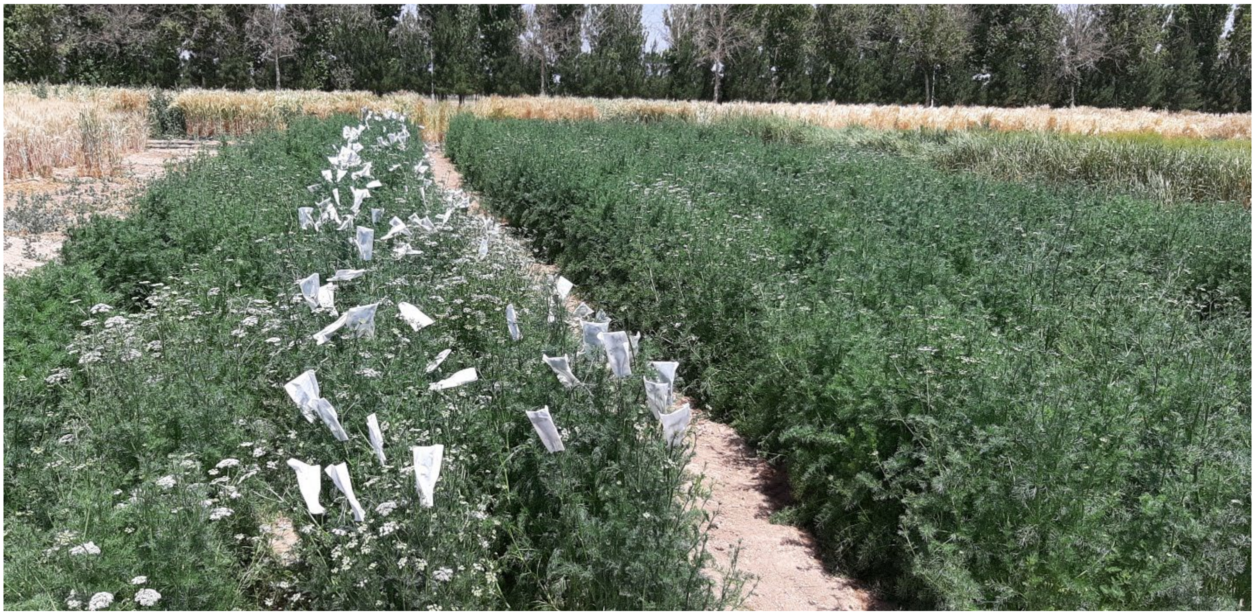

(**c**)

Figure S1. (**a**) Twenty-eight populations of ajowan originated from Iran and three populations originated from the Leibniz Institute of Plant Genetics and Crop Plant Research (IPK), Gatersleben, Germany were sown in a randomized complete block design (RCBD) in under field conditions at Lavark Research Farm of Isfahan University of Technology in two years 2017 and 2018. (**b**) In salt stress, a pot experiment was carried out under greenhouse conditions with an average temperature of 25 °C and an average humidity of 50%. Each pot contained nine kilograms of soil at a soil to sand ratio of 3:1. (**c**) In 2018, plant of each populations possessed half of its umbels bagged from the start of inflorescence emergence until seed harvest for oblige selfing, whereas the other half were left uncovered to enable open-pollination.
